# Susan Lindquist: a tribute

**DOI:** 10.1242/dmm.028696

**Published:** 2017-01-01

**Authors:** Vivian Siegel

**Figure:**
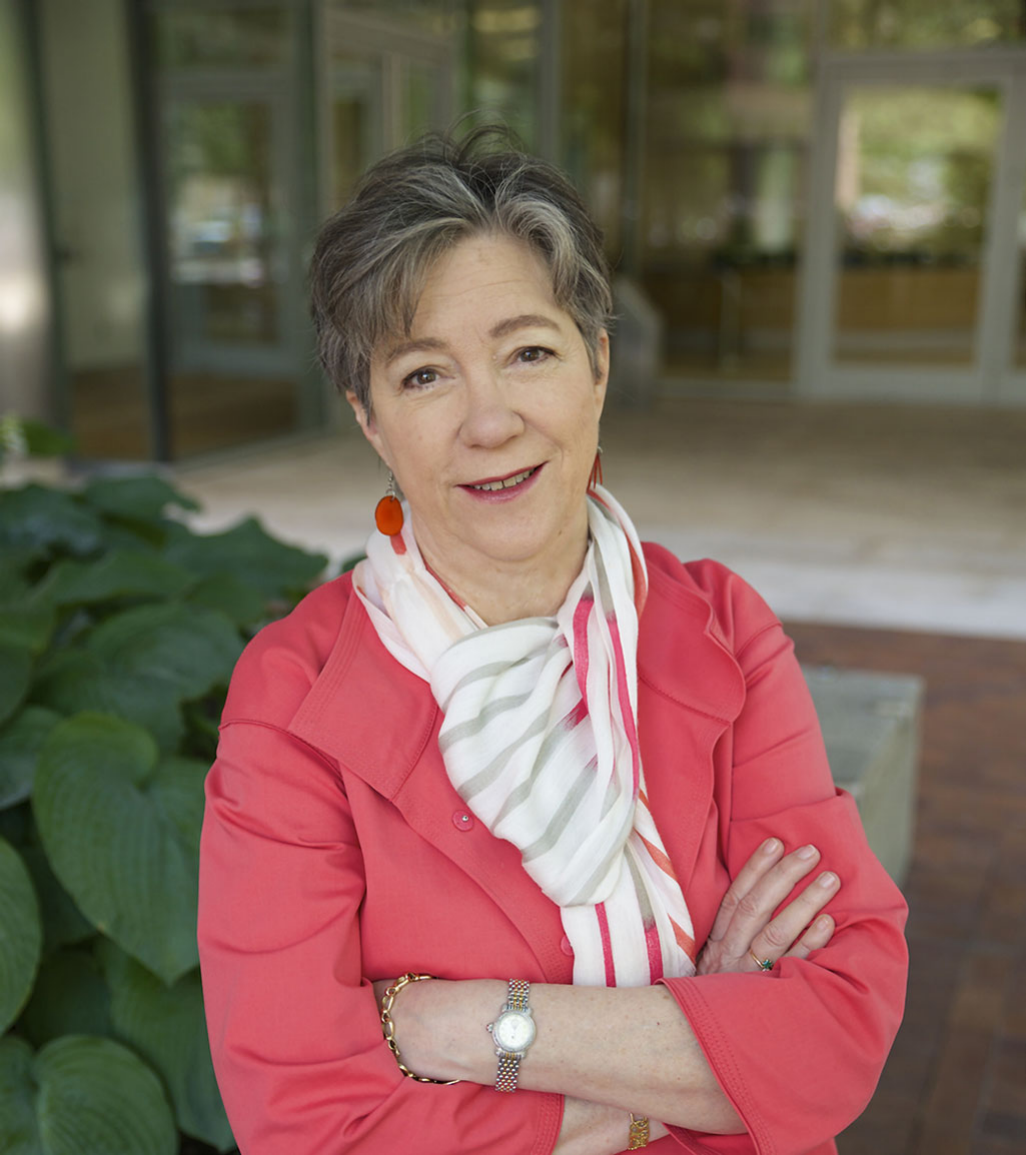
**Photo by Ceal Capistrano, Whitehead Institute.**

When I learned that Susan Lindquist had passed away at the age of 67 from cancer, my heart broke. This fierce flame for science and for women had been extinguished, and for a moment the world became a little dimmer.

I first met Susan Lindquist in 1994 when I was a postdoc interviewing for a faculty position at the University of Chicago. We met for breakfast at a local diner. We talked about our science and she impressed upon me how supportive an environment the University of Chicago was for female scientists, how they were there for each other, cheering each other's success, knowing that any time one of them succeeded, it became easier for all of them to succeed. And shortly after I returned home from that visit, I received a handwritten note from her, telling me how much she had liked the talk I gave about my research and how much she hoped I would make her university my home. She made me feel that I had what it took, that I could take this path if I wanted to, and that she and other women would have my back and help me thrive in an academic setting.

And just like that, Susan Lindquist became a lifelong mentor.

I've never worked in her field, and yet she made herself available to me time and again as I embarked upon different projects, giving me her feedback, supporting me however she could. She supported me when I helped launch Public Library of Science (PLOS), hosting me to give a seminar at the Whitehead Institute, where she was then Director; and she supported me when I launched Disease Models & Mechanisms, providing critical guidance as one of its founding editors and even hosting a meeting that led to a special issue on protein-folding diseases (Brodsky, 2014). The issue included a poster review that was co-authored by Susan and is DMM's most-read poster published to date (Valastyan and Lindquist, 2014).

It's no surprise DMM interviewed Susan for the first article in its ‘Model for Life’ section (Lindquist, 2008). In that article, she spoke about the criteria she used to select new members of her laboratory: “In terms of my own laboratory, I have two types of criteria. First, that people be bright and creative and rigorous…. Good scientists. But every bit as important, a very high priority in bringing someone in is knowing that they are generous, open, want to share information, like to help other people, and are willing and ready to accept advice from other people.”

Born in Chicago, Susan was a first-generation college student at University of Illinois at Urbana-Champaign, a graduate student at Harvard, and a postdoc at the University of Chicago, where she established her first lab. After 23 years at the University of Chicago, in 2001 she became the first woman to direct the Whitehead Institute, when she also joined the biology department faculty at MIT. She stepped down as Director of the Whitehead Institute in 2004 and remained a Whitehead Institute member, an associate member of the Broad Institute of MIT and Harvard, an associate member of the David H. Koch Institute for Integrative Cancer Research, and a biology professor at MIT for the rest of her career.

**Figure:**
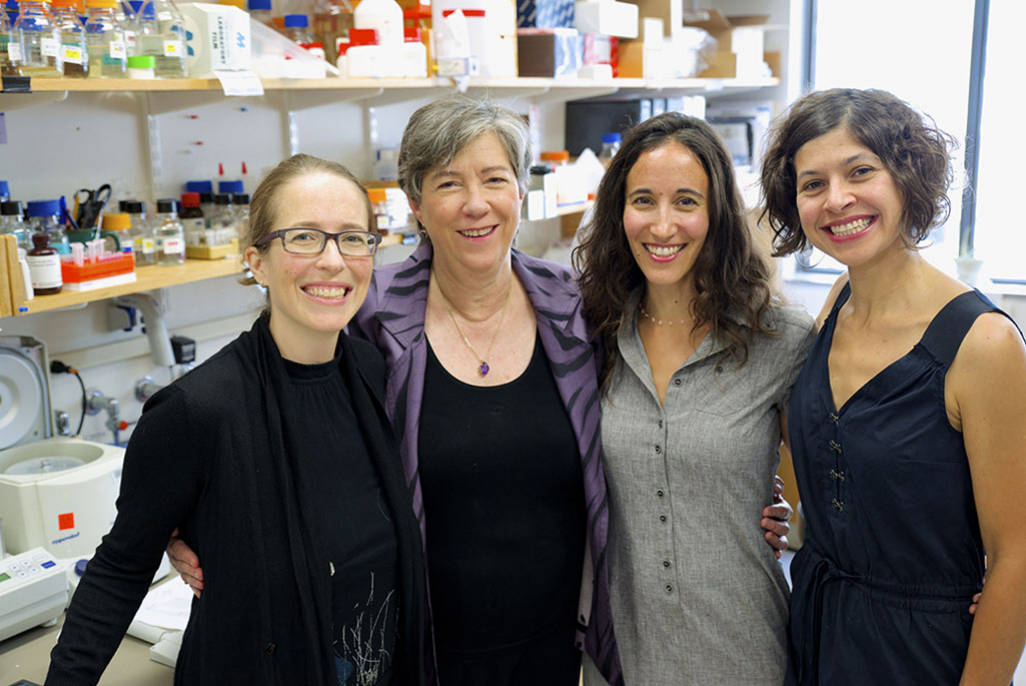
**Susan Lindquist with former post-docs Kendra Frederick, Ruth Scherz-Shouval and Gabriela Caraveo Piso. Photo by Ceal Capistrano, Whitehead Institute.**

Other obituaries focus more extensively on her science, which was spectacular by any standard. I got to hear during that breakfast meeting about her studies with PSI and Sup35 and the support her work provided for the prion hypothesis (Patino et al., 1996). This work was the beginning of what became a major focus for the rest of Susan's career: using yeast as a way to elucidate the biology of protein folding, and to understand and treat protein-folding diseases, including both neurodegeneration and cancer. She has published numerous significant papers in this field, including several papers within weeks of her death (Chakrabortee et al., 2016; Landgraf et al., 2016; Vincent et al., 2016). In brief, Susan has used yeast as a ‘living test tube’ to recapitulate many of the diseases of protein folding and to screen for drugs that might treat these diseases. To hear Susan lecture about her work, go to iBiology (https://www.ibiology.org/ibioseminars/protein-folding-infectious-disease-cancer.html).

Some recollections will highlight her awards and honors, and there were many, including, to name just a few, membership in the National Academy of Sciences, the National Academy of Medicine, and the Royal Society; the Genetics Society of America Medal, the EB Wilson Medal of the American Society of Cell Biology, the Otto Warbug medal, the Max Delbruck medal, and the National Medal of Science. For the National Medal of Science, Susan was cited “for her studies of protein folding, demonstrating that alternative protein conformations and aggregations can have profound and unexpected biological influences, facilitating insights in fields as wide-ranging as human disease, evolution, and biomaterials.”

Still others will point out her entrepreneurship and her deep passion to leverage basic science discoveries to tackle diseases linked to abnormal protein folding. In addition to being a professor at MIT and a Howard Hughes Medical Investigator, Susan founded or cofounded several companies including FoldRX Pharmaceuticals (now a wholly owned subsidiary of Pfizer Inc.) and Yumanity Therapeutics, and served on the board of Johnson & Johnson, where she chaired its Science, Technology and Sustainability Committee.

But for me, the focus has to be on Susan as an exemplary mentor, as a fierce advocate for science and for women in science, and as a friend. Aptly, Johnson & Johnson recently endowed the Susan Lindquist Chair for Women in Science at the Whitehead Institute, to be awarded to a distinguished female scientist who is advancing biomedical research. Susan demonstrated that it is possible to perform spectacular research, garner your share of accolades (although some would suggest she was overdue for a Nobel Prize), and still support the people around you – your family, your colleagues, your students, and your friends. She made it clear that you can live in a web of collegiality and connection where each success lifts everyone up, and for that example, I will be eternally grateful.

And for those of us whose lives have been forever changed by knowing Susan, we know too that her flame burns in each of us, and that we honor her memory with our support for each other, for the next generation, and for science itself.

Footnote: Donations in Susan's honor can be directed to the Whitehead Institute Fund to Encourage Women in Science. Programmatic efforts supported by this fund will complement the new Lindquist Professor's mentoring and public outreach efforts, and may include K-12 programs, a lecture series, or a symposium for women in science (https://donations.wi.mit.edu/lindquistfund).
